# Sex-specific role of epigenetic modification of a leptin upstream enhancer in adipose tissue

**DOI:** 10.1186/s13148-025-01830-2

**Published:** 2025-02-11

**Authors:** Luise Müller, Rebecca Oelkrug, Jens Mittag, Anne Hoffmann, Adhideb Ghosh, Falko Noé, Christian Wolfrum, Esther Guiu Jurado, Nora Klöting, Arne Dietrich, Matthias Blüher, Peter Kovacs, Kerstin Krause, Maria Keller

**Affiliations:** 1 Medical Department III – Endocrinology, Nephrology, Rheumatology, University of Leipzig Medical Center, 04103 Leipzig, Germany; 2 Institute for Experimental Endocrinology - Center of Brain Behavior and Metabolism (CBBM), University of Lübeck, 23562 Lübeck, Germany; 3 Helmholtz Institute for Metabolic, Obesity and Vascular Research (HI-MAG) of the Helmholtz Center Munich at the University of Leipzig and University Hospital Leipzig, Phillip-Rosenthal Str. 27, 04103 Leipzig, Germany; 4 Institute of Food, Nutrition and Health, ETH Zurich, 8092 Schwerzenbach, Switzerland; 5 Department of Visceral, Transplantation, Thoracic and Vascular Surgery, Section of Bariatric Surgery, University Hospital Leipzig, 04103 Leipzig, Germany; 6 Deutsches Zentrum für Diabetesforschung E.V., 85764 Neuherberg, Germany

**Keywords:** Maternal thyroid hormones, Body fat, Leptin upstream enhancer, DNA methylation, Sex specific, Adipose tissue, Epigenetic editing

## Abstract

**Objective:**

Maternal hormonal status can have long-term effects on offspring metabolic health and is likely regulated via epigenetic mechanisms. We elucidated the effects of maternal thyroid hormones on the epigenetic regulation of *leptin* (*Lep*) transcription in adipose tissue (AT) and subsequently investigated the role of DNA methylation at a *Lep* upstream enhancer (UE) in adipocyte biology.

**Results:**

Pregnant mice treated with triiodothyronine (T3) produced offspring with reduced body weight, total fat mass, and gonadal white adipose tissue (gWAT) mass at 6 months of age (treatment: *N* = 8; control: *N* = 12). Compared with control offspring, exclusively female offspring of T3-treated mothers presented lower *Lep* mRNA levels and higher *Lep* UE methylation in gWAT. In murine preadipocytes, targeted demethylation of the *Lep* UE via a dCas9-SunTag-TET1 system reduced methylation by ~ 20%, but this effect was insufficient to alter *Lep* expression or lipid accumulation after differentiation. In human omental visceral AT (OVAT) samples from the Leipzig Obesity BioBank (LOBB, *N* = 52), *LEP* UE methylation was associated with body fat percentage, and mediation analysis indicated that leptin serum levels partially mediate this association exclusively in females.

**Conclusion:**

Findings from the animal model suggest that maternal thyroid hormones influence offspring gWAT *Lep* expression in a sex-specific manner, potentially through changes in *Lep* UE methylation. However, in vitro experiments indicate that *Lep* UE methylation alone is not sufficient to regulate *Lep* expression or adipocyte lipid accumulation. In humans with obesity, *LEP* UE methylation is associated with body fat percentage, with leptin serum levels potentially acting as a mediator exclusively in females.

**Supplementary Information:**

The online version contains supplementary material available at 10.1186/s13148-025-01830-2.

## Introduction

Obesity is defined as an abnormal or excessive accumulation of body fat. To prevent and treat obesity, it is important to understand the regulators of body weight and fat mass. One key master regulator of body weight is the hormone leptin, which is secreted primarily from white AT. This adipokine is crucial in energy homeostasis and metabolism playing a significant role in obesity [1, 2]. Many studies of leptin function have focused on the hormone's action in the brain via its receptors, whereas the regulatory elements that enable dynamic changes in *leptin* gene expression have received less attention. *Leptin* transcription can be influenced by various factors, such as insulin [3], hypoxia [4], and hormonal signals [5]. Moreover, a fat-specific long non-coding RNA (lncOb), which is regulated alongside fat mass and influences *leptin* expression by interacting with redundant enhancers [6, 7], has been described. In mice, the absence of functional lncOb is linked to lower plasma leptin levels and increased adiposity, but these mice still respond to leptin treatment [6]. In humans, a single-nucleotide polymorphism (rs10487505) in the corresponding region is associated with circulating leptin levels and obesity in a sex-specific manner [8] but not with *leptin* gene expression, suggesting post-transcriptional mechanisms, e.g. the regulation of long non-coding RNAs [9].

According to the developmental origins of health and disease (DOHaD) theory, the development of adipose tissue and the risk of developing obesity and metabolic diseases later in life are influenced by pre- and perinatal environmental exposures in utero [10–15]. Research has shown that maternal factors during pregnancy can significantly alter the hormonal environment of the developing foetus, leading to lasting effects on *Lep* regulation in offspring [13, 16–20]. For instance, maternal high-fat diet exposure is linked to sustained increases in leptin levels and elevated blood pressure in offspring, which are mediated by epigenetic memory [20]. Maternal nutrition during pregnancy can modify the relationship between leptin levels, body fat, and caloric intake in offspring, leading to excess adiposity and metabolic issues in adulthood [17].

Thyroid hormones are known to be significant regulators of foetal tissue development and maturation [21]. Thyroid deficiency before birth alters adipose tissue development, leading to overgrowth of white adipocytes, disrupted thermogenesis, and changes in gene expression related to metabolism and insulin resistance, potentially increasing the risk of neonatal survival issues, obesity, and metabolic dysfunction later in life [22]. While the essential role of maternal thyroid hormones in foetal development is well established, the effects of maternal hyperthyroidism on offspring remain poorly understood. A recent study in mice by Oelkrug et al. demonstrated that maternal T3 treatment during gestation leads to improved glucose tolerance in adult male offspring and hyperactivity of brown adipose tissue thermogenesis in both sexes starting early after birth [23]. However, the precise mechanisms through which thyroid hormones influence foetal adipose tissue development remain unclear. Interestingly, elevated levels of choline, which is involved in the synthesis of S-adenosylmethionine, a major methyl donor required for DNA methylation, were detected in the serum of T3-treated dams [23]. This finding suggests that epigenetic mechanisms may play a role in mediating the effects of thyroid hormones on foetal adipose tissue development.

Epigenetic mechanisms, including DNA methylation, are believed to mediate the impact of prenatal stress and other factors on obesity risk in children [11, 24, 25]. Pre- and perinatal environmental exposures, including diet and pollutants, can induce epigenetic changes that impact disease development in children [25]. Maternal intake of methyl-group donors can affect offspring health by altering DNA methylation patterns, linking early environmental exposure to disease risk in offspring [26]. Furthermore, Lecoutre et al. showed that maternal obesity might epigenetically programme increased *Lep* gene expression via the modulation of upstream enhancer DNA methylation in offspring, contributing to white adipose tissue accumulation [16]. This analysed enhancer is located around 36 kb upstream from the leptin gene and in close proximity to the long non-coding RNA lncOb.

Taken together, pre- and perinatal exposure to environmental factors can significantly influence the risk of developing obesity and metabolic diseases later in life. Among these factors, maternal thyroid hormone levels during pregnancy have emerged as critical determinants of offspring metabolic health. Therefore, this study aims to elucidate the effects of maternal thyroid hormone levels on *Lep* expression in offspring, focusing on whether these effects are mediated through epigenetic regulation, specifically DNA methylation of the *leptin* upstream enhancer (*Lep* UE). Furthermore, this research explores whether epigenetic editing at this upstream enhancer can modulate *Lep* expression and adipogenesis in vitro. Finally, we examine the relationship between DNA methylation of the human *LEP* UE in OVAT, leptin expression, and body fat percentage.

## Materials and methods

### Animal model

**Fig. 1:**
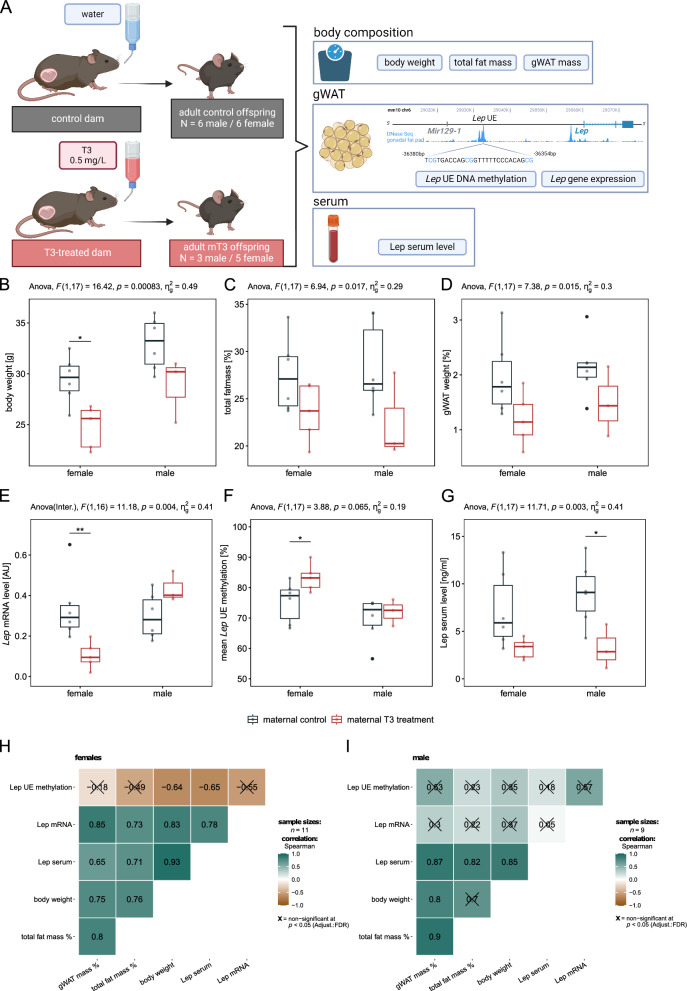
Effects of high maternal thyroid hormone during gestation on murine adipose tissue biology in offspring and *Leptin* mRNA expression. **A** Scheme of experimental design. Genomic region plot was adapted from epigenome browser (http://epigenomegateway.wustl.edu/browser/) including information about DNase sequencing data of gonadal white adipose tissue (gWAT). Location of the analysed target sequence including three CpG sites in the analysed *Lep* enhancer (~ 36 kb upstream of the transcription start site of *Lep)* is shown. Scheme was generated using biorender.com. **B**–**G** Boxplots show sex-specific alterations in **B** body weight [g], **C** total fat mass [% body weight], **D** gonadal fat mass [% body weight], as well as **E** *Lep* mRNA level in gWAT analysed by quantitative real-time PCR and normalised to the level of *Rplp0*, **F** average DNA methylation level [%] of *Lep* upstream enhancer across all three analysed CpG sites by bisulphite pyrosequencing in gWAT from offspring, and **G** leptin serum level (ng/ml) determined by ELISA for adult offspring (5–7 months) of T3-treated dams (mT3, in red, *N* = 5 females, *N* = 3 males) compared to offspring from control dams (in grey, *N* = 6 females, *N* = 6 males). Biological replicates were obtained from three litters per group. Simple main effect of maternal T3 treatment calculated by two-way ANOVA (sex + treatment) is reported on top of the graphs. For Lep mRNA level (**E**), the results of a two-way interaction ANOVA (sex * treatment) are displayed. Significance levels of pairwise comparisons calculated by unpaired Wilcoxon rank-sum test are shown for **P < 0.01 and *P < 0.05. **H/I** Heatmaps show Spearman correlation coefficients of enhancer DNA methylation, mRNA level, and serum levels of leptin with anthropometric phenotypes for **H** female offspring (mT3: *N* = 5, control: *N* = 6) and **I** male offspring (mT3: *N* = 3, control:* N* = 6). Positive correlations are shown in petrol and negative correlations in brown. Non-significant correlations (FDR < 0.05) are crossed out

This study analysed the effect of maternal hyperthyroidism on white adipose tissue function in offspring using a subset of samples from a previously published study in mice by Oelkrug et al. [23]. A scheme of the experimental design is depicted in Fig. 1A. Hyperthyroidism during pregnancy was induced in wild-type female C57BL/6NCrl (Charles River, Germany) at the age of 3–4 months with 0.5 mg/L T3 (3,3′,5-triiodo-L-thyronine, T6397; Sigma-Aldrich/Merck, Germany) in the drinking water with 0.01% BSA from the day of positive plug check until gestational day 18 (= day before birth). The hyperthyroidism of mothers was confirmed by elevated serum T3 levels, lower serum T4 levels, and completely suppressed hypophyseal thyroid-stimulating hormone β (*Tshb*) mRNA expression, as well as elevated gene expression of hepatic deiodinase type I (*Dio1*) and thyroid hormone-inducible hepatic protein (*Thrsp*) in treated dams [23]. We ensured that biological replicates were obtained from three litters per group. The body composition of offspring was analysed using Minispec LF110 and Minispec Plus Software 6.0 (Bruker, USA) at the age of 5–7 months. Serum was collected after two centrifugation steps at 4 °C (1000×*g*) and stored at − 20 °C. Adipose tissue samples were collected on the day of sacrifice (male offspring: 5–6 months and female offspring: 6–7 months), weighed, snap frozen on dry ice, and stored at − 80 °C until nucleic acid extraction. All animal experiments and procedures were approved by the Ministerium für Energiewende, Klimaschutz, Umwelt und Natur MEKUN Schleswig–Holstein, Germany.

### Human cohort

Human OVAT samples from the Leipzig Obesity BioBank (LOBB; https://www.helmholtz-munich.de/en/hi-mag/cohort/leipzig-obesity-bio-bank-lobb) were collected from 518 individuals with severe obesity (median BMI [IQR] = 48.1 [41.9–53.7] kg/m^2^) at the university hospital in Leipzig. For DNA methylation analysis of the *LEP* UE, a subset of 52 individuals (female: *N* = 31, male: *N* = 21) with a median BMI [IQR] = 49.0 [41.5–52.6] kg/m^2^ was used. OVAT samples were collected during elective laparoscopic abdominal surgery as previously described [27, 28], immediately frozen, and stored at − 80 °C. The exclusion criteria encompassed participants under 18 years of age, chronic substance or alcohol misuse, smoking within the 12 months prior to surgery, acute inflammatory diseases, the use of glitazones as concomitant medication, end-stage malignant diseases, weight loss exceeding 3% in the 3 months preceding surgery, uncontrolled thyroid disorder, and Cushing's disease. All participants gave written informed consent before taking part in the study and were informed of the purpose, risks, and benefits of the biobank. The study was approved by the ethics committee of the University of Leipzig (#159-12-21052012).

Phenotyping of the LOBB includes assessment of age, sex (self-reported), BMI (kg/m^2^), body fat mass (%) detection via bioelectrical impedance analysis, and metabolic biochemical assessment. Leptin serum levels were determined by enzyme-linked immunosorbent assay (ELISA) (Leptin Human ELISA, Mediagnost, Germany) as previously described [8]. *Leptin* mRNA levels were extracted from RNA-sequencing data, which is described in detail in the Suppl. Information. The phenotypic characteristics of the total cohort as well as the subset used for methylation analysis are found in Table 1.

**Table 1:** Phenotypic characteristics of LOBB cohort

	Cohort	N	Median [IQR]	Female		Male		Female vs. male
				*N*	Median [IQR]	*N*	Median [IQR]	*P*-value
BMI [kg/m^2^]	Total	518	48.1 [41.9–53.7]	339	47.1 [41.8–52.7]	179	50.0 [42.6–54.7]	0.065
	Methylation subset	52	49.0 [41.5–52.6]	31	46.5 [39.6–51.0]	21	52.0 [47.7–57.6]	**0.031**
Age [y]	Total	518	50.4 [40.1–57.0]	339	50.5 [40.8–57.0]	179	50.1 [39.2–57.2]	0.716
	Methylation subset	52	50.0 [39.3–56.8]	31	54.0 [40.9–57.4]	21	48.3 [35.2–53.5]	0.376
Body fat [%]	Total	511	48.8 [41.9–54.3]	337	52.0 [47.0–57.1]	174	41.8 [37.0–46.8]	**6.19E−33**
	Methylation subset	52	47.3 [40.6–55.8]	31	54.5 [42.0–58.7]	21	44.8 [39.8–48.0]	**0.017**
TSH [mU/l]	Total	510	1.6 [1.0–2.2]	335	1.5 [0.9–2.2]	175	1.7 [1.1–2.2]	0.072
	Methylation subset	52	1.9 [1.2–2.4]	31	1.8 [1.2–2.4]	21	1.9 [1.2–2.4]	0.963
fT3 [pmol/l]	Total	327	4.9 [4.3–5.4]	212	4.8 [4.2–5.3]	115	5.2 [4.5–5.6]	**0.002**
	Methylation subset	46	5.0 [4.7–5.4]	27	5.0 [4.6–5.3]	19	5.2 [4.7–5.6]	0.289
fT4 [pmol/l]	Total	338	16.6 [14.6–18.9]	222	17.0 [14.8–19.4]	116	15.6 [14.5–18.1]	**0.011**
	Methylation subset	48	17.0 [14.9–19.7]	29	18.4 [15.8–21.6]	19	15.8 [14.8–17.5]	0.058
*LEP* mRNA level [AU]	Total	518	12.2 [11.5–13.0]	339	12.1 [11.4–12.9]	179	12.5 [11.6–13.2]	**0.045**
	Methylation subset	52	12.3 [11.4–13.0]	31	12.3 [11.3–12.9]	21	12.7 [11.5–13.9]	0.274
LEP serum level [ng/µl]	Total	485	40.2 [25.8–54.8]	315	41.9 [30.4–57.8]	170	31.2 [17.9–47.9]	**4.70E−07**
	Methylation subset	50	41.6 [35.9–50.4]	30	43.4 [36.4–51.3]	20	41.1 [34.9–47.3]	0.434
*LEP* UE meth. [%]*	Methylation subset	52	63.5 [56.3–67.7]	31	65.3 [60.8–68.5]	21	59.1 [53.2–65.5]	**0.031**

Data are shown as median with interquartile range [Q1–Q3]. Comparisons between female and male were calculated by unpaired Wilcoxon rank-sum test. Significant *P*-values (< 0.05) were marked in bold

*AU* arbitrary units, *BMI* body mass index, *TSH* thyroid-stimulating hormone, *fT3* free triiodothyronine, *fT4* free thyroxine, *LEP* leptin, and *UE* upstream enhancer

*Mean DNA methylation of 6 CpG sites

### Plasmid preparation for in vitro hypomethylation

Targeted in vitro hypomethylation was performed as described by Morita et al. using the pPlatTET-gRNA2 vector, which was a gift from Izuho Hatada (Addgene plasmid #82559; http://n2t.net/addgene:82559; RRID:Addgene_82559) [29]. This all-in-one plasmid contains the dCas9-SunTag and single-chain variable fragment (scFv)-TET1 catalytic domain (TET1CD) system, and a gRNA expression system. Three different gRNAs targeting the murine *Lep* UE were designed using the CRISPR website from Dr. Feng Zhang’s laboratory (http://crispor.tefor.net) [30]. Cloning was performed by linearisation of the pPlatTET-gRNA2 with Afl II (NEB, USA) and Gibson assembly mediated incorporation of the gRNA insert fragment as described previously [31]. The target sequences are listed in Suppl. Table 1.

### In vitro Lep UE hypomethylation

We used SV40 T-antigen immortalised murine preadipocyte cells isolated from the epididymal white adipose tissue of C57BL6 mice [32], which were grown at 37 °C in a 5% CO_2_ atmosphere in Dulbecco's Modified Eagle's Medium (DMEM)-high glucose (Gibco/Thermo Fisher Scientific, USA) supplemented with 10% Foetal Bovine Serum (FBS Superior S0615, Sigma-Aldrich/Merck, Germany). The cells were transfected with 5 µg of the all-in-one plasmids (1.67 µg for each gRNA) via electroporation using the Neon™ Transfection System (Invitrogen/Thermo Fisher Scientific, USA). To increase the transfection efficiency of large-size CRISPR/Cas9 vectors [33], cells were co-transfected with the equimolar amount of an empty pGEM®-T vector (Promega, USA) with a size of approximately 3 kb. As a negative control, cells were transfected with pPlatTET-gRNA2 without incorporated gRNA (plasmid control). The cells were sorted 24 h post-transfection for GFP-positive cells using FACS Aria II (BD Biosciences, USA) and the BD FACSDiva Software 9.0.1 (BD Biosciences, USA) and seeded back in growth media supplemented with 1% penicillin/streptomycin to avoid potential contamination. The differentiation of cells is described in detail in the Suppl. Information.

### Murine leptin ELISA

Mouse leptin ELISA (Cat # 90,030, CrystalChem, USA) was performed according to the manufacturer’s instructions. In brief, 5 µl of sample or standard was added to each well and incubated overnight at 4 °C. The optical density at 450/630 nm was measured using the SPECTROstar Nano Microplate Reader (BMG Labtech, Germany).

### Bisulphite sequencing of human and murine *leptin* upstream enhancer

Genomic DNA extraction from murine gWAT, human OVAT, and from cultured adipocytes was performed as described in detail in the Suppl. Information. DNA was bisulfite converted using the EpiTect Fast Bisulfite Conversion Kit (Qiagen, Germany) and further amplified using the EpiTect Whole Bisulfitome Kit (Qiagen, Germany). The target sequences were amplified using self-designed primers (Suppl. Table 1), and pyrosequencing was performed using the PyroMark Q24 technologies and corresponding Gold Kits (Qiagen, Germany). Non-template controls were included in all steps to rule out contamination. All PCR products were subjected to quality control using agarose gel electrophoresis prior to sequencing. Only the methylation values of CpG positions with good quality (automatic blue calling) were considered for analysis. All the experiments were performed in duplicate.

### Quantitative RT-PCR analysis

The RNA extraction procedure is described in detail in the Suppl. Information. The cDNA synthesis from murine gWAT RNA was performed using QuantiTect Reverse Transcription Kit (Qiagen, Germany) with integrated removal of genomic DNA contamination and SuperScript III (Invitrogen/Thermo Fisher Scientific, USA) for RNA from cell culture samples. A non-enzyme control, as well as a non-target control without RNA, was always carried along to control for genomic DNA and other contaminants. Quantitative real-time PCR was performed using Power Up SYBR Green (Applied Biosystems/Thermo Fisher Scientific, USA) in the LightCycler 480 System (Roche, Switzerland). Self-designed primers are found in Suppl. Table 1. Measurement of a standard curve with a serial dilution of pooled cDNA was included to determine the efficiency of the PCR. Relative quantities of mRNA were determined using the efficiency-based ΔCt method normalised to the mRNA level of *Rplp0* [34]. The measurements were performed in triplicate and averaged.

### Gene expression profiling of murine gWAT via Clariom S arrays

Gene expression profiling of gWAT samples from offspring of maternal T3 treatment experiment was performed at the Core Facility for DNA technologies of Knut Krohn from the University of Leipzig via GeneChip Clariom S arrays (Affymetrix/Thermo Fisher Scientific, USA). The raw microarray data were preprocessed using the oligo R package (v1.50.0), which includes background correction and quantile normalisation through the Robust Multichip Average (RMA) algorithm [35, 36]. Further quality control was carried out using both the Biobase (v2.46) and oligo R packages [37]. Genes with median transcript intensities below a threshold of 4 were filtered out from the normalised dataset. Differentially expressed genes (DEGs) were identified using the Linear Models for Microarray Data method in the R package limma (v3.42) [38], incorporating array weights to enhance the signal-to-noise ratio. The thresholds for the identification of DEGs were an unadjusted *P*-value < 0.01 and a |log2-fold change (FC)|≥ 0.5.

### Lipid staining and quantification using AdipoRed™ assay

Intracellular lipid accumulation was assessed by fluorescence spectroscopy after staining with AdipoRed™ reagent (Lonza, Switzerland). Successfully transfected cells were grown in 96-well plates and differentiated as described in the Suppl. Information. At days 0, 4, and 8 of differentiation, the intracellular lipids were stained with AdipoRed™ (1:40 in PBS, 10 min) and measured using a FLUOstar OPTIMA Microplate Reader (BMG Labtech, Germany; excitation 485 nm; emission 520 nm). DNA was then stained with Hoechst 33,342 (Sigma-Aldrich/Merck, Germany; 1:2000 in PBS, 15 min), and the fluorescence intensity was measured (excitation 355 nm; emission 460 nm) to estimate cell number. The relative lipid amount was calculated as the ratio of AdipoRed™ fluorescence intensity to Hoechst fluorescence intensity. Using the same staining procedure, microscopy of the cells was performed using a Carl Zeiss Axio Observer Z1 (Carl Zeiss, Germany) with the filter set 38 HE green and ZEN software. Fluorescence images were captured using the FITC channel for lipids and the DAPI channel for DNA.

### Statistics and bioinformatic analysis

Statistical analyses were performed using R v.4.4.1. The effects of maternal T3 treatment on offspring phenotypes (body weight, total fat mass, and gWAT mass), leptin serum levels, *Lep* mRNA expression, and DNA methylation of the *Lep* UE were tested via two-way ANOVA, with sex and maternal treatment as factors. To address potential violations of using ANOVA (e.g. non-normality), we complemented the analysis with non-parametric tests (Wilcoxon rank-sum) for post hoc comparisons, ensuring robust interpretation of the results, and defining *P* < 0.05 as significant. Spearman’s rank correlation was performed to assess associations between continuous variables using *ggcormat* from the R package *ggstatsplot* v.0.12.4. The effects of in vitro hypomethylation treatment on *Lep* UE DNA methylation, *Lep* mRNA levels, and lipid accumulation during the course of adipocyte differentiation were analysed via two-way mixed ANOVA, using time as the within factor and treatment as the between factors. Simple main effects were further analysed by pairwise paired Student’s t-test. Overrepresentation analysis for Wikipathways with the gene lists, which were found to be differentially expressed between offspring gWAT of T3-treated vs. control dams, was performed using *clusterProfiler* v.4.12.2 [39]. The analysis was performed separately for upregulated (log2 FC > 0, *P* < 0.05) and downregulated genes (log2 FC < 0, *P* < 0.05). Causal mediation analysis was conducted to address two assumptions: i) Whether the observed sex-specific differences in body fat percentage are mediated via LEP UE DNA methylation in human OVAT, and ii) whether the relationship between female-specific LEP UE DNA methylation and body fat percentage is mediated by leptin serum levels. The mediation analyses were performed using *mediation* R package v.4.5.0. We employed bootstrapping procedures to test the significance of the indirect effects, i.e. we generated 1000 bootstrapped samples to estimate the unstandardised indirect effects. The 95% confidence intervals for the indirect effects were computed by determining the 2.5th and 97.5th percentiles of the bootstrapped indirect effects.

## Results

### Offspring body composition after maternal T3 treatment

Two-way ANOVA revealed a significant main effect of maternal T3 treatment during pregnancy on body weight (g), total fat mass, and gWAT mass relative to body weight (%) in adult offspring (*P* < 0.02, Fig. 1B-D). Subsequent pairwise comparisons stratified by sex revealed a significant effect on body weight specifically in female offspring (*P* = 0.017, Fig. 1B), with similar trends observed for both sexes and fat mass. Apart from this, serum levels of thyroid hormones (T3, T4, and TSH) were not altered in offspring of T3-treated dams compared with control offspring [23], which is in agreement with the findings of similar previous studies [40, 41].

### Leptin expression and methylation levels in offspring after maternal T3 treatment

We observed a significant interaction effect between sex and maternal T3 treatment on *Lep* mRNA levels in offspring gWAT (F(1,16) = 11.18, *P* = 0.004, Fig. 1E), suggesting a sex-specific effect of maternal T3 treatment on *Lep* mRNA levels in the offspring. While significantly lower *Lep* mRNA levels were detected in the female offspring of T3-treated mothers than in the control offspring (*P* = 0.009, Fig. 1E), the *Lep* mRNA levels in the gWAT of the male offspring born to the T3-treated dams were, albeit higher, not significantly different (*P* = 0.167; Fig. 1E) from that of the control dams.

The gWAT DNA methylation at the previously reported enhancer region ~ 36 kb upstream of *Lep* revealed no significant interaction effect of sex and T3 treatment. However, DNA methylation was significantly higher in the female offspring of the T3-treated mothers (median methylation difference = 5.9%, *P* = 0.03; Fig. 1F) than in those of the control animals. No differential methylation was detected between male offspring born to T3-treated and control dams (median methylation difference = 0.3%, *P* = 0.714; Fig. 1F). For leptin serum levels in offspring, no significant interaction effect of sex and T3 treatment was found, but a simple main effect of T3 treatment was evident (*P* = 0.003; Fig. 1G). After stratification by sex, the difference was significant only for the male offspring (*P* = 0.034; Fig. 1G), but for the female offspring of the T3-treated mothers, a trend towards lower leptin serum levels was still observed (*P* = 0.058, Fig. 1G) than that of the controls.

Irrespective of maternal treatment, we observed a strong positive correlation of *Lep* mRNA expression in gWAT as well as leptin serum levels with gWAT mass (% of body weight), total fat mass (% of body weight), and body weight (Spearman rho > 0.6, FDR < 0.05; Fig. 1H) in female offspring. In male offspring, we observed this positive correlation only for leptin serum levels with gWAT mass (% of body weight), total fat mass (% of body weight), and body weight (Spearman rho > 0.8, FDR < 0.05; Fig. 1I), whereas *Lep* mRNA expression in gWAT did not correlate with any trait. In female offspring, *Lep* UE methylation was negatively correlated with body weight and leptin serum levels (Spearman rho < − 0.6, FDR < 0.05; Fig. 1H) but not significantly correlated with *Lep* mRNA expression in female offspring. In male offspring, *Lep* UE DNA methylation in gWAT did not correlate with any trait at all (all FDRs > 0.05; Fig. 1I).

### Genome-wide expression changes in offspring gWAT after maternal T3 treatment

To further explore the molecular determinants of altered body composition in offspring of the T3-treated vs. control dams, we performed microarray gene expression analysis in gWAT of adult offspring. While the effect sizes of maternal T3 treatment on the adult offspring might not be sufficient to detect genome-wide significant differences in gene expression after correction for multiple testing, we identified several DEGs using relaxed cut-offs with |log2 FC|> 0.5 and an unadjusted *P* < 0.01 (females: 291 DEGs, Suppl. Table 2; males: 862 DEGs, Suppl. Table 3). A summary of the most significant DEGs can be found in volcano plots (Suppl. Figure 1). Notably, *Lep* was among the most significantly altered genes in female offspring (log2 FC = -0.84, *P* = 0.0026), which was consistent with our quantitative real-time PCR results. To gain further insight, we performed a Wikipathway overrepresentation analysis on upregulated and downregulated genes in adult offspring following maternal T3 treatment. The analysis of the female offspring revealed that upregulated genes (*Creb1*, *Foxo1*, *Gata2*, *Nr2f2*, *Rora*, *Klf5)* are involved in ‘white fat cell differentiation’ (*WP2872*, FDR = 0.048; Suppl. Figure 2A, Suppl. Table 4). Additionally, six (*Mvk*, *Dhcr7*, *Lss*, *Idi1*, *Hmgcr*, and *Sqle*) of the fifteen genes of the ‘cholesterol biosynthesis’ pathway (*WP103*, FDR = 0.001) were among the downregulated genes in the female offspring of T3-treated dams compared with those of the control animals (Suppl. Figure 2A, Suppl. Table 4). In gWAT of male offspring, this analysis revealed that gene expression of 20 out of 268 ‘non-odorant G-Protein coupled receptors’ (GPCRs) was upregulated (*WP1396*, FDR = 0.035), whereas the expression of 16 genes from 81 encoding ‘cytoplasmic ribosomal proteins’ (*WP 163*, FDR = 2 × 10^–6^) was downregulated in offspring of the T3-treated dams compared with those of the control dams (Suppl. Figure 2B, Suppl. Table 5).

### In vitro hypomethylation of Lep upstream enhancer using a dCas9-Suntag-TET1 system

**Fig. 2:**
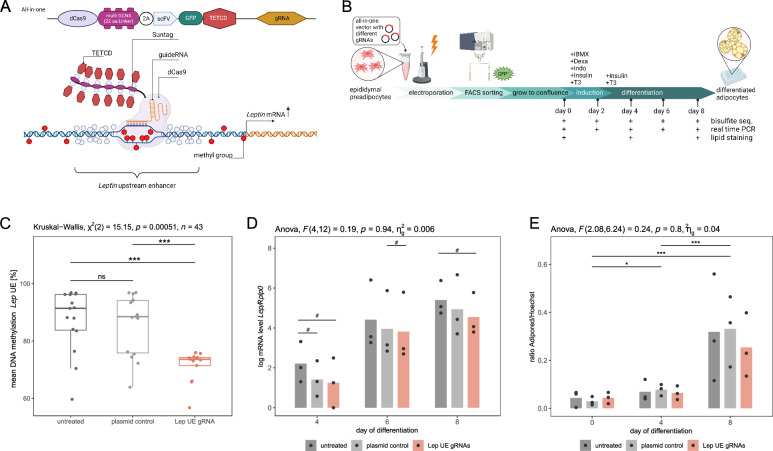
In vitro* Lep* UE hypomethylation and effect on adipocyte differentiation. **A** Principal scheme of targeted in vitro hypomethylation using a dCas9-Suntag-TET1 system showing in the top the ‘all in one’ vector which contains dCas9 peptide array (linker length: 22 amino acids), antibody-sfGFP-TET1CD, and gRNA expression system. Scheme was generated using biorender.com. **B** Workflow of the experiment, which is described in detail in the methods section. For the hypomethylation, epididymal preadipocytes were transfected by electroporation with all-in-one vectors including three different gRNAs targeting the *Lep* UE (= Lep UE gRNA, red). For control, we used untreated cells (= UT, dark grey) and cells transfected with the all-in-one vector without gRNA (= plasmid control, light grey). Workflow was generated using biorender.com. **C** Boxplot shows DNA methylation level [%] of *Lep* UE across all three analysed CpG sites by bisulphite pyrosequencing. Significance of differences between treatments was calculated using Kruskal–Wallis test, followed by pairwise comparisons by Wilcoxon rank-sum test corrected for multiple testing by FDR (***FDR < 0.001, ns FDR > 0.05). Dots represent mean methylation levels of days 0, 2, 4, 6, and 8 of differentiation for each experiment (*n* = 3 experiments × 5 time points). **D** Barplot shows the mean of log transformed *Lep* mRNA levels normalised to *Rplp0* mRNA levels for days 4, 6, and 8 of adipocyte differentiation from *n* = 3 experiments. Results of mixed two-way ANOVA used to assess the effect of treatment and time on *Lep* expression are shown on top of the graph. Pairwise comparisons of treatment effects using paired Student's t-test (paired by experiment number) are depicted in the graph. No significant differences were found after correction for multiple testing by FDR. Shown significance level are uncorrected for multiple testing: #*P* < 0.05. **E** Barplot shows the lipid amounts in cells during differentiation on days 0, 4, and 8 between treatments. Shown is the average ratio of Adipored and Hoechst fluorescence intensity from *n* = 12 wells in *n* = 3 experiments. Results of mixed two-way ANOVA used to assess the effect of treatment and time on lipid accumulation are shown on top of the graph. Significance levels from pairwise comparison of time points using paired Student's t-test (paired by experiment number) and corrected for multiple testing by FDR are depicted in the graph (***FDR < 0.001, **FDR < 0.01, and *FDR < 0.05). No significant differences are found when comparing treatments

To investigate the functional role of DNA methylation at the *Lep* UE in vitro*,* the target region was hypomethylated in immortalised murine epididymal preadipocytes using a system consisting of a dCas9–SunTag and scFv–TET1 catalytic domain (Fig. 2A). The workflow of the experiment is illustrated in Fig. 2B. After transfection, we achieved an approximately 20% reduction of *Lep* UE DNA methylation in successfully transfected cells (mean DNA methylation ± SD = 71.0 ± 4.6%) compared with that in untreated cells (mean DNA methylation ± SD = 90.4 ± 6.4%) on the day of induction (= day 0). We further observed no significant increase in DNA methylation during adipocyte differentiation in the transfected cells (Suppl. Figure 3), indicating a stable reduction in DNA methylation during differentiation. After combining DNA methylation at all time points, we observed significantly different DNA methylation rates between the treatments (*P* = 5.1 × 10^–4^, Fig. 2C), and subsequent pairwise comparisons confirmed significantly lower DNA methylation in the cells transfected with the vector targeting the *Lep* UE (median DNA methylation [IQR] = 73.6 [71.4–74.3] %) than in the untreated cells (median DNA methylation [IQR] = 91.4 [83.8–96.2] %, FDR < 0.001) and the cells transfected with the plasmid control without gRNA (median DNA methylation [IQR] = 88.5 [75.9–94.1] %, FDR < 0.001). We observed no significant difference in DNA methylation between the cells transfected with the plasmid control and the untreated cells (FDR = 0.87), indicating that there was no significant global effect due to increased amounts of the TET1 catalytic domain per se.

### Effects of upstream enhancer hypomethylation on *Lep* mRNA levels during adipocyte differentiation in epididymal preadipocytes

The effects of UE hypomethylation on the *Lep* mRNA level and lipid accumulation during differentiation were evaluated. Since *Lep* mRNA levels were not detectable on the day of induction or 2 days after induction (Ct values > 33), only the mRNA levels on day 4 and later were considered. No significant interaction effect of treatment and time on the *Lep* mRNA level was detected (ANOVA, F(4,12) = 0.19, *P* = 0.94; Fig. 2D). A simple main effect of time on the *Lep* mRNA level was found, indicating that the *Lep* mRNA level increased over the course of adipocyte differentiation (ANOVA, F(2,12) = 110.119, *P* = 1.9 × 10^–8^). We did not observe a significant effect of treatment on the *Lep* mRNA level (ANOVA, F(2,12) = 0.309, *P* = 0.745). After pairwise comparisons, we observed nominal lower *Lep* mRNA levels in the transfected cells than in the untreated cells at days 4 and 8 (*P* < 0.05, Fig. 2D). However, this trend of lower *Lep* expression was also observed in the cells transfected with the plasmid control compared to untreated cells (*P* < 0.05). On day 6, we observed slightly lower *Lep* expression in treated cells (Lep UE gRNAs) than in plasmid control cells (*P* < 0.05). The observed differences were moderate, and none of these comparisons were statistically significant different after correction for multiple testing. Overall, *Lep* expression remained low even after differentiation (day 8 Ct values > 28). Consistently, leptin secretion into the cell culture medium was minimal, making ELISA measurements unreliable and not robustly evaluable.

### Effects of upstream enhancer hypomethylation on lipid accumulation during adipocyte differentiation in epididymal preadipocytes

The effect of *Lep* UE hypomethylation on lipid accumulation during adipocyte differentiation was assessed via AdipoRed staining of lipids. No significant interaction effect on the lipid accumulation was detected between hypomethylation and time (ANOVA, F(2.08, 6.24) = 0.24, *P* = 0.8; Fig. 2E). A significant increase of lipids during the course of adipocyte differentiation (ANOVA, F(1.04,6.24) = 23.998, *P* = 0.002) was confirmed by pairwise comparisons (FDR < 0.05, Fig. 2E), which supported the accumulation of lipids during differentiation. However, no significant difference was detected in cells after hypomethylation of the *Lep* UE compared with the control cells (Fig. 2E).

### Effects of in vitro hypomethylation of the *Lep* UE in other adipocyte cell lines

Additionally, we performed experiments in 3T3L1 cells as well as in immortalised female cells from the inguinal (subcutaneous) fat depot. In 3T3L1 cells, we achieved on average a 35% hypomethylation of the *Lep* UE in transfected vs. untreated cells (Suppl. Figure 4A). In these cells, on day 4, a significant difference in the *Lep* mRNA levels was detected between hypomethylated and untreated cells (FDR < 0.01; Suppl. Figure 4B), but not compared with that in the plasmid control cells, and this difference was not detected in the following days of differentiation. Additionally, in 3T3L1 cells, we observed no effect of *Lep* UE hypomethylation on lipid accumulation (Suppl. Figure 4C). In the inguinal cells from female mice, on average 21% hypomethylation of *Lep* UE (transfected vs. untreated inguinal cells, Suppl. Figure 5A) was observed at all time points. Although we were not able to detect *Lep* mRNA expression despite successful adipocyte differentiation, we observed a trend towards reduced lipid accumulation after hypomethylation of the *Lep* UE in these cells (Suppl. Figure 5B/C).

### Sex-specific differences in leptin expression and methylation levels in human visceral adipose tissue

Bisulphite sequencing of a corresponding *LEP* UE region in human OVAT (*N* = 52) revealed significantly higher methylation levels in female (*N* = 31, median methylation [IQR] = 65.3 [60.8–68.5] %) than in male individuals (*N* = 21, median methylation [IQR] = 59.1 [53.2–65.5] %, *P* = 0.031, Table 1). In the entire cohort, in female subjects, we observed a significantly lower OVAT *LEP* mRNA expression (*N* = 518, *P* = 0.045, Table 1), whereas leptin serum levels were significantly higher (*N* = 485, *P* < 0.0001, Table 1) than in males.

### Sex-specific causal relationship of human *LEP* upstream enhancer methylation with body fat percentage

**Fig. 3:**
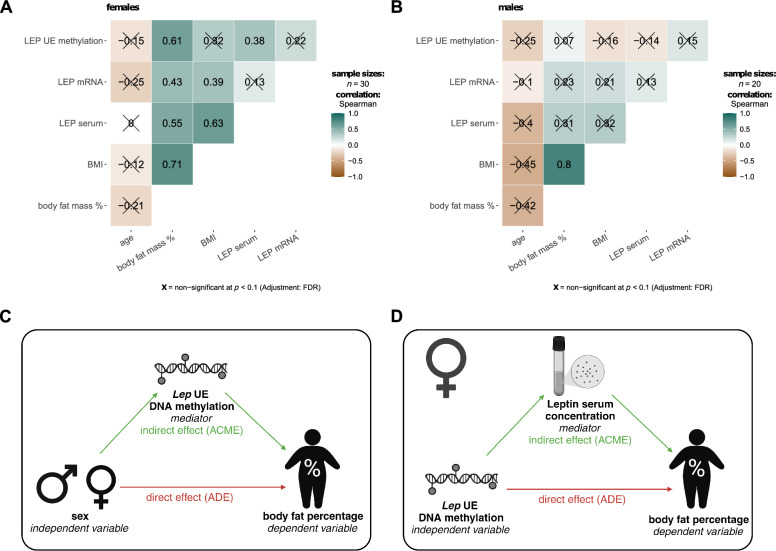
Relationship between sex-specific differences in leptin expression and enhancer DNA methylation levels in human visceral adipose tissue with body fat percentage. Heatmaps show Spearman correlation coefficients of enhancer DNA methylation, mRNA level, and serum levels of leptin with anthropometric phenotypes for female (**A**) and male (**B**) individuals with obesity. Positive correlations are shown in petrol and negative correlations in brown. Non-significant correlations (FDR < 0.1) are crossed out. **C** Scheme for causal mediation analysis exploring the mediation of sex-specific differences in body fat percentage via DNA methylation at the *LEP* upstream enhancer. Results of the mediation analysis are shown in Table 2. **D** Scheme for causal mediation analysis in female individuals exploring the mediation of the effect of DNA methylation at the *LEP* upstream enhancer on body fat percentage via leptin serum level. Results of the analysis are shown in Table 3

Correlation analysis with DNA methylation at the *LEP* upstream enhancer revealed a significant positive correlation with body fat (% of body weight) in female individuals with obesity (Spearman rho = 0.61, FDR < 0.05; Fig. 3A). We also observed a positive correlation between *LEP* methylation and leptin serum levels in female individuals (Spearman rho = 0.38, FDR < 0.1; Fig. 3A). We found no significant association between *LEP* UE methylation and *LEP* mRNA level (FDR > 0.1). In male individuals with obesity, no correlation of the *LEP* UE methylation with any trait was identified (all FDRs > 0.1; Fig. 3B). Additionally, *LEP* mRNA levels in OVAT and leptin serum levels correlated with no other trait in males. Since *LEP* UE methylation did not correlate with *LEP* mRNA expression in OVAT, we addressed another causal relationship of DNA methylation with body fat percentage. First, we tested in a mediation analysis whether *LEP* UE DNA methylation functions as a mediator for the sex-specific difference in body fat percentage (Fig. 3C, Table 2). In our model, the sex-specific effect on body fat percentage was fully mediated via DNA methylation at three of the six analysed CpG sites (CpG 4–6: ADE *P* > 0.1, ACME *P* < 0.05; Table 2) and partially mediated at CpG site 1 (ADE *P* < 0.1, ACME *P* < 0.05). The percentage of mediation ranged from 30.8% (CpG site 1) to 48.1% (CpG site 4). Since *LEP* UE methylation correlated with the body fat percentage only in females, we further used a second mediation analysis to test whether this association was mediated via the leptin serum level in females (Fig. 3D, Table 3). The second mediation analysis revealed that for DNA methylation at CpG sites 1, 4, and 6 as well as for the mean DNA methylation across all analysed CpG sites, the effect on body fat percentage was partially mediated via the leptin serum level (ADE *P* < 0.01, ACME *P* < 0.05; Table 3). The percentage of the mediation effect ranged from 27.6% (CpG site 6) to 32.5% (CpG site 4).

**Table 2:** Causal mediation analysis of sex > *LEP* UE methylation > body fat percentage

CpG site	*a*	*b*	*c*	ACME	ADE	Total effect	Prop. mediated
1	− 3.956	0.6150**	− 7.891*	− 2.43293*	− 5.45805	− 7.89098**	0.3083
2	− 2.895	0.3584*	− 7.891*	− 1.038	− 6.853*	− 7.891**	0.131
3	− 3.037	0.3255	− 7.891*	− 0.989	− 6.902*	− 7.891*	0.125
4	− 5.636*	0.6490*	− 7.891*	− 3.6580*	− 39.533	− 7.6113*	0.4806*
5	− 6.282*	0.4904*	− 7.891*	− 3.0810*	− 48.100	− 7.891**	0.3904*
6	− 4.497	0.7128***	− 7.891*	− 3.2053*	− 46.857	− 7.8910**	0.4062*
Mean	− 4.213	0.5719**	− 7.891*	− 2.4095	− 5.4815	− 7.8910**	0.3053

Significance level: ***P < 0.001; **P < 0.01; *P < 0.05; and *P* < 0.1; regression coefficients: a—between sex and DNA methylation, b—between DNA methylation and body fat (corrected for sex effect), and c—direct effect between sex and body fat

ACME: average causal mediation effects = indirect effect of sex on body fat that goes through the mediator (DNA methylation); ADE: average direct effects = direct effect of sex on body fat when controlling for the mediator (DNA methylation); Total effect: direct + indirect effect of sex onto body fat (ACME + ADE); and Prop. mediated: proportion of sex on body fat that goes through the mediator (DNA methylation)

**Table 3:** Causal mediation analysis of *LEP* UE methylation > LEP serum level > body fat percentage (in female individuals)

CpG site	*a*	*b*	*c*	ACME	ADE	Total effect	Prop. mediated
1	0.9296	0.29736***	0.9141**	0.2764**	0.6377**	0.9141***	0.3024**
2	0.5334	0.31914***	0.5347*	0.1702	0.3645**	0.5347**	0.3183
3	0.7282	0.30786***	0.6012**	0.2242	0.3770*	0.6012*	0.3729
4	1.327**	0.23764**	0.9708***	0.3155***	0.6554**	0.9708***	0.3249***
5	0.7471	0.29057***	0.7869***	0.2171	0.5698***	0.7869***	0.2759
6	1.0253*	0.26327***	0.9759***	0.2699*	0.7060***	0.9759***	0.2766*
Mean	1.0448*	0.2721***	0.9456***	0.2843**	0.6613**	0.9456***	0.3007**

Significance level: ***P < 0.001; **P < 0.01; *P < 0.05; and *P* < 0.1; regression coefficients: a—between LEP DNA methylation and LEP serum level in female individuals, b—between LEP serum level and body fat (corrected for DNA methylation effect), and c—direct effect between DNA methylation and body fat

ACME: average causal mediation effects = indirect effect of DNA methylation on body fat that goes through the mediator (LEP serum level), ADE: average direct effects = direct effect of DNA methylation on body fat when controlling for the mediator (LEP serum level), Total effect: direct + indirect effect of DNA methylation onto body fat (ACME + ADE), and Prop. mediated: proportion of DNA methylation on body fat that goes through the mediator (LEP serum level)

## Discussion

This study provides novel insights into the possible role of maternal thyroid hormones in shaping offspring metabolic outcomes by analysing DNA methylation and gene expression in the gWAT of adult offspring after maternal T3 treatment during pregnancy. Our findings underscore the importance of maternal thyroid hormones during pregnancy in influencing adult offspring body composition and provide insights into the sex-specific influence of DNA methylation at the *Lep* UE on leptin expression and body fat percentage.

In the present study, maternal T3 treatment resulted in reduced body weight, total fat mass, and gWAT mass in adult offspring of both sexes. The increased DNA methylation at the *Lep* UE and the corresponding decrease in *Lep* mRNA expression in female offspring indicated that high T3 exposure in utero might epigenetically programme *Lep* expression, potentially altering adipose tissue function and reducing adiposity. In contrast, male offspring did not exhibit significant changes in *Lep* UE methylation but did show altered leptin serum levels, indicating potential sex-specific differences in the downstream effects of maternal T3-induced metabolic changes. A possible mechanism involves the differential expression and activity of thyroid hormone receptors and enzymes in the placenta and foetal tissues. Studies indicate that the placental deiodinase, which regulates T3 availability, is differentially expressed based on foetal sex [42], potentially leading to variations in metabolic programming with maternal T3 still influencing offspring in a sex-specific manner.

Additional findings support the hypothesis, that the observed effects in offspring are more likely a consequence of metabolic changes in the mothers induced by elevated T3 levels. Oelkrug et al. described the effects of maternal T3 treatment, including increased weight gain during pregnancy, increased food and water intake, and improved glucose clearance. While offspring from T3-treated dams presented lower body weights in adulthood (5–6 months), Oelkrug et al. reported higher embryonal and birth weights in these animals than in controls. They also reported no significant differences in body weights between offspring of T3-treated dams and controls during postnatal development until week 10. Taken together, these findings suggest early developmental programming with long-term effects on gene expression and metabolic outcome.

Although the findings from the genome-wide expression analysis in gWAT from offspring of T3-treated and control dams were not statistically significant after correction for multiple testing, the findings point to several differentially expressed genes, with notable involvement in pathways related to adipogenesis and lipid metabolism. In females, genes related to white fat cell differentiation were upregulated, though most inhibited differentiation (*Foxo1, Gata2, Nr2f2,* and *Rora*), suggesting potential suppression of adipogenesis and lipogenesis. Genes involved in cholesterol biosynthesis were downregulated. In males, upregulation of non-odorant GPCRs suggests potential alterations in signalling pathways linked to energy homeostasis. GPCRs are implicated in the regulation of energy expenditure through mechanisms such as thermogenesis. These findings highlight sex-specific differences in metabolic programming influenced by maternal T3 treatment.

To the best of our knowledge, this is the first study to demonstrate targeted dCas9/TET1-mediated hypomethylation in preadipocytes. We believe that a 20% reduction in DNA methylation should be sufficient to impact gene expression, since the differences seen in the gWAT of the animal model were much smaller. However, we could not establish a direct causal relationship between DNA methylation at the *Lep* upstream enhancer (UE) and *Lep* gene expression. This may reflect the complexity of gene regulation, where DNA methylation in this region is one of many factors. Other mechanisms, such as chromatin structure or transcription factor availability, might compensate for reduced methylation [43]. It is also plausible that DNA methylation in this region affects transcription of adjacent genes, such as the lncOb described by Dallner et al. [6], which might regulate post-transcriptional events rather than directly influencing *Lep* gene expression. Supporting this, Lo et al. found that lncOb knockdown impaired adipogenesis, but its overexpression had minimal effects on leptin levels or other adipocyte markers suggesting that lncOb is essential for leptin regulation but is insufficient to drive these processes independently [7].

Sex-specific regulation might also be the reason for the absence of a detectable effect of DNA hypomethylation in this region, since we used cells originating from male mice. However, the relationship of DNA methylation at this region with leptin expression or body fat regulation was mainly observed in females. Our experiment in female inguinal cells suggested that hypomethylation of the upstream enhancer region affected lipid accumulation, indicating possible female-specific regulation. Sex-specific differences in in vitro leptin production and secretion in response to oestrogen have already been shown for adipocytes from female vs. male sources [44]. However, since *Lep* expression was not detectable in female inguinal cells, the mechanistic explanation for the role of DNA methylation in lipid accumulation remains unclear.

Furthermore, in vitro cell culture conditions may not be conducive to cultivating leptin expressing adipocytes. The evidence suggests the existence of different types of adipocytes, each characterised by distinct gene expression patterns, with only certain adipocytes expressing leptin [45]. Moreover, adipocytes are influenced in vivo by various cell types such as macrophages, endothelial cells, fibroblasts, and neurons. These cells release cytokines, growth factors, and hormones that regulate adipocyte function, including leptin expression. In vitro, this network of cellular crosstalk is missing or incomplete. Therefore, in vitro models can mimic some aspects of adipocyte biology, but they often fail to fully replicate the conditions needed for proper leptin expression. To further explore the role of this enhancer region in the regulation of leptin expression, it might be necessary to use a cell culture system that involved the interaction of different cell types, such as spheroids, or to analyse the epigenetic modifications via in vivo models.

Our data revealed a significant sex-specific difference in *LEP* UE DNA methylation in OVAT from individuals with severe obesity, with females exhibiting higher DNA methylation levels than males. Moreover, female subjects presented lower mRNA expression levels but higher leptin serum levels than males did. The higher serum levels of leptin in women are well documented and are attributed not only to a greater proportion of adipose tissue but also to a higher production rate of leptin per unit mass of adipose tissue [46–48]. Furthermore, the influence of sex hormones, particularly oestrogens, has been suggested to modulate leptin expression, although the exact mechanisms remain complex and are not fully understood [49, 50]. In terms of fat depot specificity, leptin expression varies significantly across different adipose tissue depots. Studies have shown that *LEP* mRNA levels are generally higher in subcutaneous fat than in visceral fat depots, such as omental fat [51–53]. The higher mRNA expression of *LEP* in the OVAT of men despite lower serum levels might be due to a lower AT mass or a greater contribution of subcutaneous AT to leptin serum levels.

Despite missing significant correlations between *LEP* UE DNA methylation and *LEP* mRNA expression in human OVAT, we identified correlations with body fat percentage and leptin serum levels, exclusively in females. Using mediation analysis, we could show a significant mediation of the sex-specific variation in body fat percentage via *LEP* UE DNA methylation. Additionally, a second analysis in females revealed that the correlation between *LEP* UE DNA methylation and body fat percentage may be partially mediated by leptin serum levels. The mechanism by which DNA methylation affects body fat percentage and leptin serum levels, despite the absence of a significant effect on gene expression, remains unclear. However, post-transcriptional regulation of leptin expression via long non-coding RNAs could explain this observation. The reported leptin-lowering effect of the lncOb rs10487505 polymorphism, without any change in *LEP* mRNA expression [8] supports this hypothesis.

Several limitations warrant cautious interpretation of the findings. The small sample size in both the animal and human studies limit the generalisability of findings and may have reduced statistical power, particularly in identifying subtle or complex interactions. In the mouse model, we ensured to use biological replicates from at least three litters per group, which increases reliability of our results. However, larger cohorts are needed to confirm the relationship between maternal T3 levels and offspring leptin expression, as well as sex-specific DNA methylation and leptin gene expression associations. While the controlled animal model enabled detailed investigation into how elevated T3 levels during pregnancy influence offspring metabolic phenotypes, such studies cannot be ethically conducted in human cohorts. Additionally, interspecies differences in adipose tissue function, hormone regulation, and fat distribution pose challenges for translating these results to humans. In this study, human adipose tissue samples were obtained during bariatric surgeries, which currently presents the most feasible way to obtain such tissue for research purposes. As a result, the findings of our study are specific to individuals with obesity—a condition often associated with leptin resistance—limiting their applicability to the broader population. This resistance might have obscured potential correlations between leptin mRNA expression and leptin serum levels. Moreover, the lack of conservation in the exact region, which was analysed in mice and humans, further complicates direct comparisons. However, both analysed regions are located inside an enhancer region, potentially governing the same function. Despite these discrepancies, a consistent finding across both the animal model and human cohort is the sex-specific association of DNA methylation at the leptin upstream enhancer. Importantly, these findings were observed exclusively in females, underscoring a potential sex-specific role for this enhancer region.

In summary, our data indicate the influence of maternal thyroid hormones on offspring gWAT *Lep* transcription in a sex-specific manner, potentially related to DNA methylation changes in the *Lep* upstream enhancer. Our human data points towards a causal relationship of a sex-specific DNA methylation effect at the human *LEP* upstream enhancer on the body fat percentage, mediated by leptin serum levels in females. The success of targeted epigenetic editing in preadipocytes might promote the use of similar approaches for the investigation of epigenetic regulations and the possible use as novel strategies in treatment of obesity and related metabolic disorders.

## Supplementary Information

Additional file 1

Additional file 2

Additional file 3

## Data Availability

The data that support the findings of this study have been deposited in public data repositories, or are presented as Supplementary Information with the manuscript. Microarray data have been deposited in the ArrayExpress database at EMBL-EBI (www.ebi.ac.uk/arrayexpress [54]) with the accession number E-MTAB-14515. The human RNA-seq data from the LOBB have not been deposited in a public repository due to restrictions imposed by patient consent but can be obtained from Matthias Blüher upon request.
